# Supplementary material for: Dose-dependent changes in global brain activity and functional connectivity following exposure to psilocybin: a BOLD MRI study in awake rats
Source: Front Neurosci. 2025 May 1;19:1554049. doi: 10.3389/fnins.2025.1554049 (PMC12078138; doi:10.3389/fnins.2025.1554049)
Supplement: Supplementary file 1 [file Table_1.docx]

| Component Name | Compound ID | Q1 Mass (Da) | Q3 Mass (Da) | Dwell Time (ms) | EP (V) | CE (V) | CXP (V) |
| --- | --- | --- | --- | --- | --- | --- | --- |
| Psilocybin | Psilocybin_240 | 285.15 | 240 | 75 | 10 | 28 | 15 |
| Psilocybin | Psilocybin_205 | 285.15 | 205.08 | 75 | 10 | 26 | 15 |
| Psilocybin | Psilocybin_164 | 285.15 | 160.03 | 75 | 10 | 43 | 12 |
| Psilocybin | Psilocybin_115 | 285.15 | 115 | 75 | 10 | 70 | 15 |
| Psilocybin | Psilocybin-d4_244 | 289.16 | 244 | 75 | 10 | 29 | 15 |
| Psilocybin | Psilocybin-d4_209 | 289.16 | 209.12 | 75 | 10 | 26 | 15 |
| Psilocybin | Psilocybin-d4_164 | 289.16 | 164.06 | 75 | 10 | 43 | 15 |
| Psilocybin | Psilocybin-d4_118 | 289.16 | 118.02 | 75 | 10 | 70 | 15 |
| Psilocin | Psilocin_160 | 205.13 | 160.05 | 75 | 10 | 30 | 10 |
| Psilocin | Psilocin_115 | 205.13 | 115 | 75 | 10 | 55 | 10 |

**Supplemental Table 1** Mass spectrometric parameter settings for the API7500. Each of the 3 compounds listed were analyzed using multiple parent (mass in Q1) and fragments (mass in Q3). Q=quadrupole; EP=exit potential, CE=collision energy, CXP=collision chamber exit potential, V=voltage.
