# Supplementary material for: Dose-dependent changes in global brain activity and functional connectivity following exposure to psilocybin: a BOLD MRI study in awake rats
Source: Front Neurosci. 2025 May 1;19:1554049. doi: 10.3389/fnins.2025.1554049 (PMC12078138; doi:10.3389/fnins.2025.1554049)
Supplement: Supplementary file 6 [file Data_Sheet_1.pdf]

Affine Transformation:

We experimented with non-linear transformations and compared them to affine registration. In clinical imaging, the complex structure of gyri and sulci often leaves no choice but to use non-linear methods—despite their known limitations. However, in preclinical imaging, we have the flexibility to evaluate whether non-linear transformations truly offer an advantage over affine registration. In our experience, we encountered two main issues: overfitting, where the transformation distorted anatomical accuracy, and significantly increased computation time, making the process far less efficient. Through this process, we learned that while non-linear registration can be a powerful tool in preclinical MRI, it also introduces several potential pitfalls that need to be carefully considered.

Typically, functional imaging data are acquired using single-shot Echo Planar Imaging (EPI) sequences due to their high temporal resolution and signal-to-noise ratio (SNR). However, in rodent imaging, these sequences frequently result in substantial geometric distortions near air-filled regions such as the sinuses at the brain's base, making linear corrections insufficient. To overcome this limitation, we employ the RARE-st pulse sequence for our fMRI data, commonly known as HASTE (Half-Fourier Acquisition Single-shot Turbo Spin Echo). The HASTE sequence is advantageous because it rapidly acquires high-quality images without the distortion characteristic of EPI sequences, enabling effective affine transformation and accurate registration.

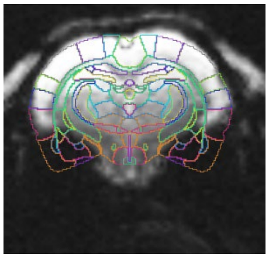	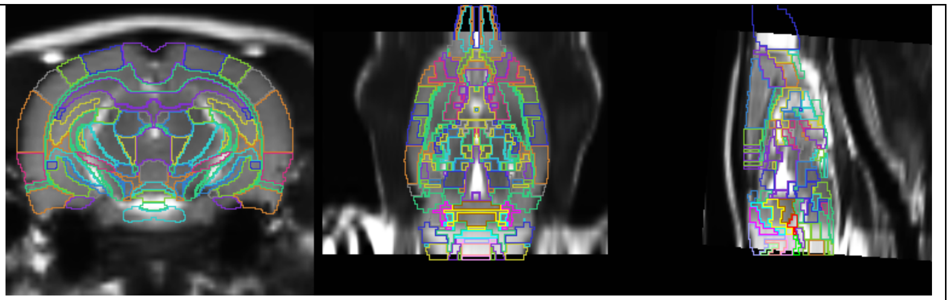
EPI sequence	HASTE sequence and registration to MRI Atlas.

Rigid body Transform:

In functional MRI (fMRI), rigid body transformation is commonly used for motion correction primarily because it effectively addresses the typical types of motion encountered during a scan, without unnecessarily complicating the correction process. Here's why rigid body transformation is particularly suited for fMRI motion correction:

1. Nature of Movements: The types of motion usually seen in fMRI studies involve translations (shifts in position) and rotations (turning around axes), which are precisely the movements that rigid body transformations can correct. These movements could be due to small head nods, tilts, or slight shifts within the scanner.
2. Preservation of Tissue Properties: Rigid body transformation maintains the integrity of the scanned tissue's properties by not altering the scale or the shape of the brain. This is crucial as any deformation beyond scaling and rotation could distort the biological structures being studied, potentially leading to inaccuracies in the interpretation of neural activity.
3. Simplicity and Efficiency: Rigid body transformations involve fewer parameters (three for rotation and three for translation), making them computationally less intensive compared to other transformation methods like affine or non-linear transformations. This efficiency is important for real-time or rapid post-processing of fMRI data.
4. Sufficiency for the Task: For most fMRI applications, correcting for simple head motions is sufficient to remove the artifacts caused by such motions, without the need for more complex modeling that could introduce other artifacts or errors.
5. Standard Practice: It has become a standard practice to use rigid body motion correction in fMRI due to its effectiveness and simplicity. Most fMRI analysis software packages provide tools that implement this method, ensuring broad compatibility and standardization across different studies and labs.
