# Supplementary material for: Dose-dependent changes in global brain activity and functional connectivity following exposure to psilocybin: a BOLD MRI study in awake rats
Source: Front Neurosci. 2025 May 1;19:1554049. doi: 10.3389/fnins.2025.1554049 (PMC12078138; doi:10.3389/fnins.2025.1554049)

**Supplemental Figure S1** Chromatograms of Psilocybin standards and plasma samples. A-B) 10 and 100 fmol Psilocybin standards injected onto the HPLC column and analyzed via HPLC/MS/MS. C-D) 10µl injections of partially purified plasma extractions analyzed using the optimized methods for Psilocybin measurements.


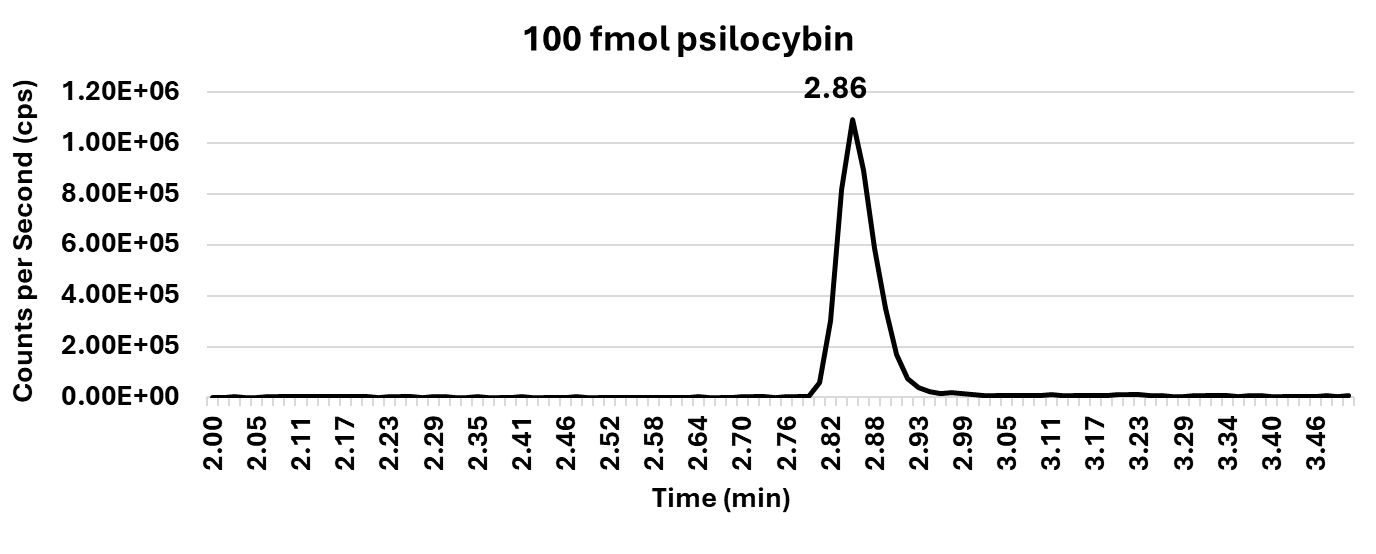


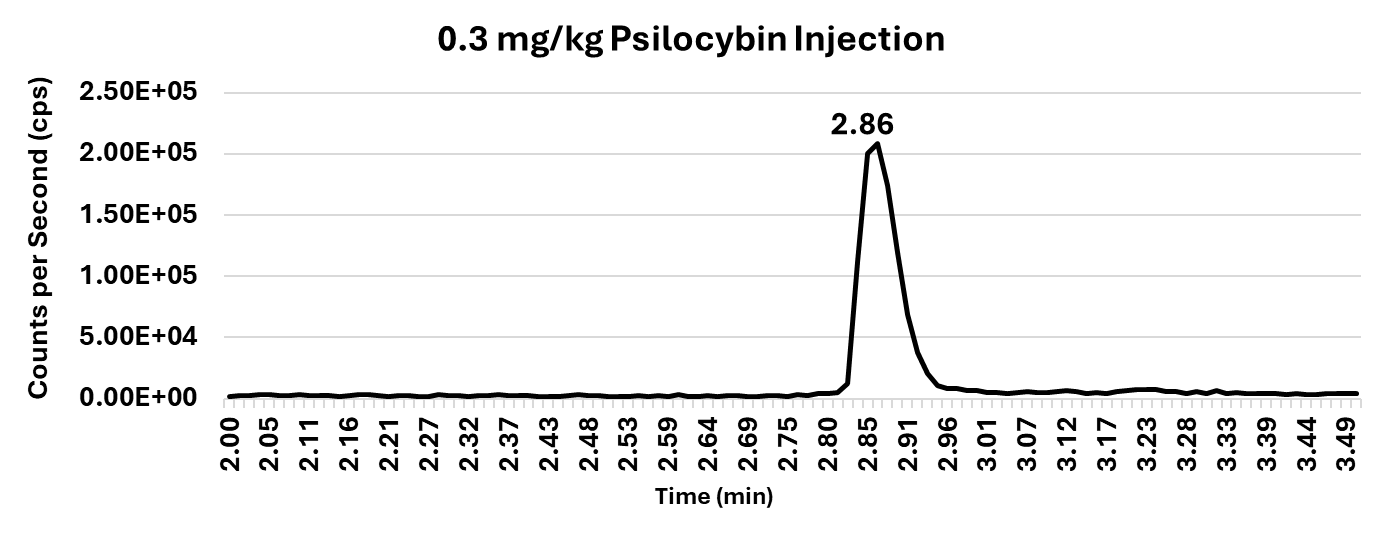


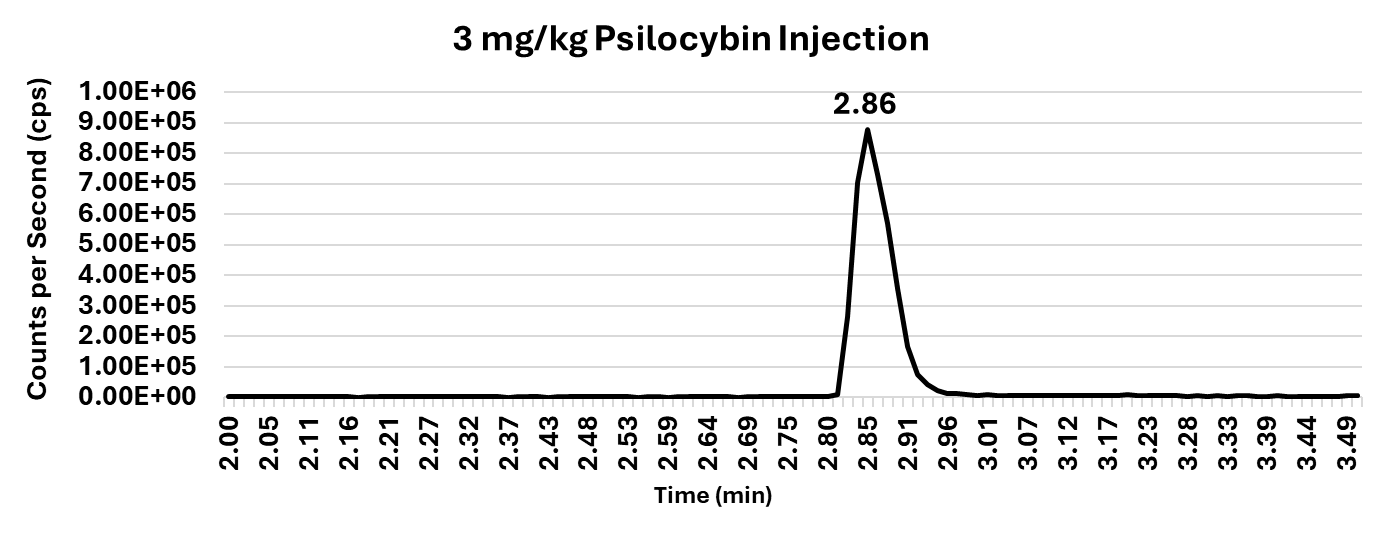


**
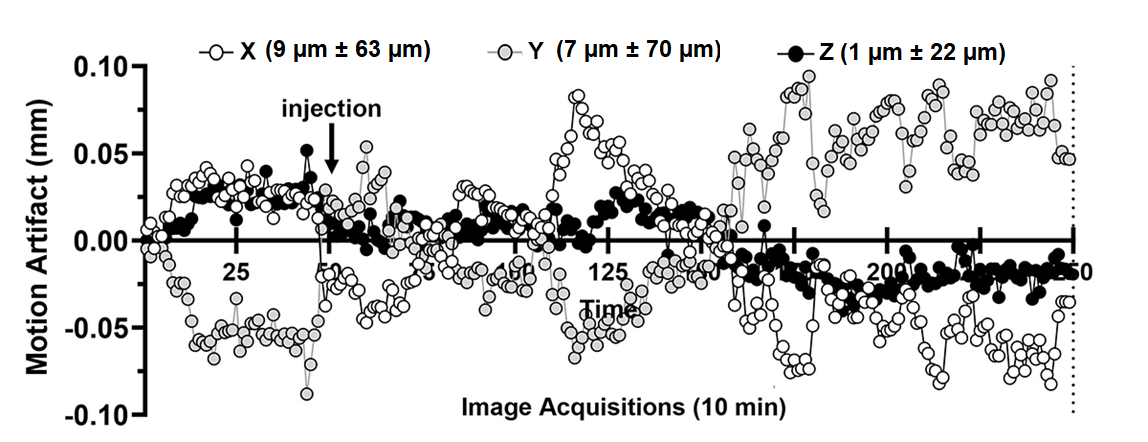
Supplementary Figure S2 – Motion Artifact**

Shown is a time course of motion artifact over the duration of the scanning protocol. The data from thirty-two rats (eight from each experimental group) were combined to show the mean ± SE for the X, Y and Z axis for 250 image acquisitions. The imaging parameters set the in-plane resolution of a pixel at was 312 µm^2^. The average motion artifact at any time point does not exceed 100 µm in any orthogonal direction.

**Supplementary Fig S3 Ketanserin time course**

Shown is the time course of BOLD signal change in the somatosensory cortices in response to 3.0 mg/kg PSI but in the presence of ketanserin. There is no significant difference between vehicle and PSI with the blockade of 5HT2 a receptors.


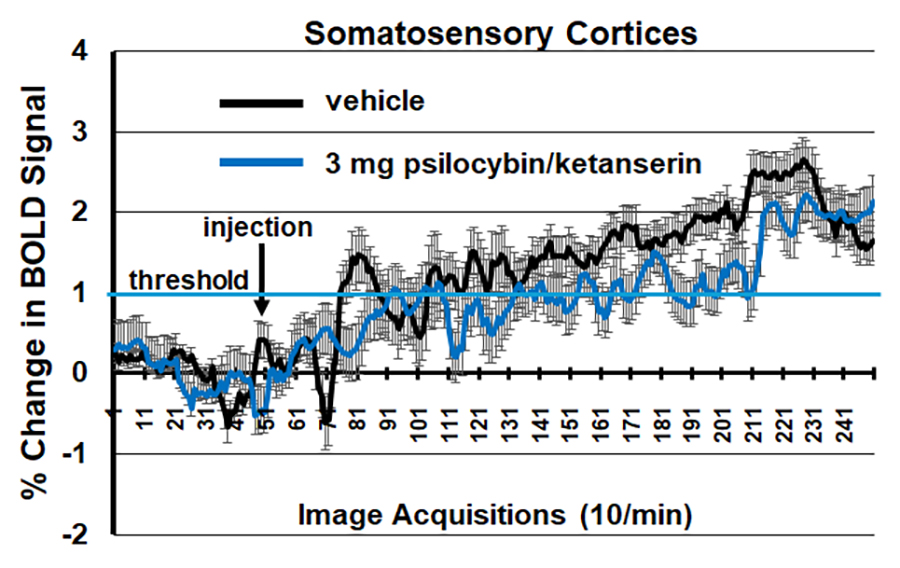

Supplement: Supplementary file 7 [file Data_Sheet_2.docx]
